# Protective role of membrane tumour necrosis factor in the host’s resistance to mycobacterial infection

**DOI:** 10.1111/j.1365-2567.2008.02865.x

**Published:** 2008-12

**Authors:** Nasiema Allie, Lena Alexopoulou, Valerie J F Quesniaux, Lizette Fick, Ksanthi Kranidioti, George Kollias, Bernhard Ryffel, Muazzam Jacobs

**Affiliations:** 1 Institute of Infectious Diseases and Molecular Medicine, University of Cape Town Cape Town, South Africa; 2 Biomedical Sciences Research Centre Alexander Fleming Vari, Greece; 3 University of Orleans, CNRS, UMR6218 Orléans, France; 4 National Health Laboratory Service Pretoria, South Africa

**Keywords:** BCG, granuloma, H37Rv, membrane Δ1-12 TNF, *Mycobacterium*, T-cell recruitment, TNF-deficiency

## Abstract

Tumour necrosis factor-α (TNF-α) plays a critical role in the recruitment and activation of mononuclear cells in mycobacterial infection. The role of membrane TNF, in host resistance against *Mycobacterium bovis* bacille Calmette–Guérin (BCG), was tested in knock-in mice in which the endogenous TNF was replaced by a non-cleavable and regulated allele (Δ1–12, TNF^tm/tm^). While 100% of mice with complete TNF deficiency (TNF^−/−^) succumbed to infection, 50% of TNF^tm/tm^ mice were able to control *M. bovis* BCG infection and survived the experimental period. Membrane expressed TNF allowed a substantial recruitment of activated T cells and macrophages with granuloma formation and expression of bactericidal inducible nitric oxide synthase (iNOS). Using virulent *Mycobacterium tuberculosis* infection we confirm that membrane TNF conferred partial protection. Infection in TNF^tm/tm^ double transgenic mice with TNF-R1 or TNF-R2 suggest protection is mediated through TNF-R2 signalling. Therefore, the data suggest that membrane-expressed TNF plays a critical role in host defence to mycobacterial infection and may partially substitute for soluble TNF.

## Introduction

Protective immunity to *Mycobacterium tuberculosis* infection, both in humans and experimental animals, is regulated by T cells, macrophages and cytokines, which include interferon-γ (IFN-γ), interleukin-12 (IL-12) and tumour necrosis factor (TNF).^1^,^2^ IFN-γ derived from T and natural killer (NK) cells has been shown to be essential, as mice with a disruption of the IFN-γ system are unable to restrict the growth of *M. tuberculosis* and succumb to the infection.^3^–^6^

A critical role for TNF in mycobacterial defence is inferred from neutralization and deletion experiments in mice.^3^–^8^ In addition, the TNF-related cytokine lymphotoxin-α, (LTα, previously known as TNF-β), has also been shown to be required to control mycobacterial infection.^9^ Secreted TNF and LTα signal both through the p55 and p75 TNF receptors, TNFR1 and TNFR2 respectively, while the cell bound LTαβ heterotrimer recognize the LTβR.^10^ TNFR1 signalling appears to be critical for the control of mycobacterial infection^5^,^8^ while a role of TNFR2 is not excluded.^11^ LTβR signalling was similarly found to protect against mycobacterial infection^12^,^13^

TNF is produced by macrophages, but is expressed by a variety of cells and is a major regulator of inflammation and leucocyte trafficking.^14^,^15^ Although a key role of TNF in controlling intracellular bacterial infections is uncontested, the function of membrane TNF, which is subsequently cleaved by the metalloproteinase-disintegrin TACE (TNF-α converting enzyme^16^) into the secreted trimeric TNF is, in mycobacterial host resistance, less clear.

Several biological functions of membrane TNF have been described, such as cytotoxicity, polyclonal activation of B cells, and induction of IL-10 by monocytes and intracellular adhesion molecule-1 expression on endothelial cells.^17^–^19^ The transgenic expression of membrane TNF demonstrated an *in vivo* role including the control of BCG and *M. tuberculosis* infection^20^–^22^ but this approach is imperfect as the transgenic expression of high levels membrane TNF is artificial and may cause altered immune responses and non-physiological pathology. As an example the spontaneous development of arthritis in transgenic mice expressing mutant membrane TNF was directly attributed to the deregulation of membrane TNF production.^23^ Two novel mutants have been generated where the endogenous TNF was either replaced by a Δ1-9,K11E TNF allele^24^ or Δ1-12 allele,^25^ which represent a major advance allowing interesting insights in the role of membrane TNF in lymphoid structure development and inflammation. We and others demonstrated that the Δ1-9,K11E mutant has enhanced resistance to *M. tuberculosis*^26^,^27^ and *Listeria monocytogenes* infection.^28^Δ1-12 TNF mutant is resistant to low-dose *L. monocytogenes* infection, but no data are available on mycobacterial infections.^25^

Here, we demonstrated that the Δ1-12 TNF mutant^25^ is partially resistant to infection with 50% of mice that succumb to the non-virulent vaccine strain *Mycobacterium bovis* BCG, while 100% of TNF-deficient mice succumb to infection. TNF^tm/tm^ mice were able to recruit and activate macrophages and T cells, and generate mycobactericidal granuloma in response to *M. bovis* BCG infection. However, resistance to the virulent *M. tuberculosis* strain was less pronounced, suggesting substantial differences between this and the Δ1-9,K11E mouse mutant.^24^

## Methods

### 

#### Animals

Adult, 8- to 10-week-old female mice of the wild type (WT) strain C57BL/6, homozygous Δ1-12 TNF mutant^25^ (TNF^tm/tm^), TNFR1^−/−^, TNFR2^−/−^ and TNF^−/−^29 strains, all on a C57BL/6 genetic background were bred and maintained under specific pathogen-free conditions. TNF^tm/tm^ mice were crossed on TNFR1^−/−^ mice^30^ or TNFR2^−/−^ mice^31^ in order to study the signalling of membrane TNF. The genotypes of the mouse populations were confirmed by polymerase chain reaction (PCR) analysis of tail biopsies. PCR primers: Δ1-12 TNF mutant – 5′-TAATGGGCAGGGCAAGGTGG-3′ and 5′-TTCACTTCCGGTTCCTGCACCCT-3′; TNFR1 – 5′-CTCTCTTGTGATCAGCACTG -3′, 5′-TCCCGCTTCAGTGACAACGTC-3′ and 5′-AGAAATCTCAAGACAATTCTCTGC-3′; TNFR2 – 5′-CCTCTCATGCTGTCCCGGAAT-3′, 5′-AGCTCCAGGCACAAGGGCGGG-3′, 5′-CGGTTCTTTTTGTCAAGAC-3′ and 5′-ATCCTCGCCGTCGGGCATGC-3′. All protocols employed in this study were approved by the University of Cape Town Research Ethics Committee.

#### Mycobacteria

*M. tuberculosis* H37Rv (Trudeau Mycobacterial Culture Collection; Trudeau Institute, Saranac Lake, NY) and *M. bovis* Bacille Calmette–Guérin (BCG) (Prof. G. Marchal, Pasteur Institute, Paris) were grown in Middlebrook 7H9 medium (Difco Laboratories, Detroit, MI), supplemented with 10% oleic acid albumin dextrose catalase (OADC) (State Vaccine Institute, Cape Town, South Africa) and 0·5% glycerol (Merck, Darmstadt, Germany) in 5% CO_2_ at 37° and frozen in aliquots. Prior to use, an aliquot was thawed, briefly vortexed, diluted in sterile saline and clumping disrupted by aspirating through a 29-gauge needle (Omnican®; Braun, Melsungen AG, Germany) 20 times.

#### Mycobacterial infection

WT, TNF^tm/tm^ and TNF-deficient mice were injected intravenously (i.v.) at an infective dose of 2 × 10^6^ BCG. Groups consisted of 10 mice and the experiments were repeated twice. Mice were killed at 2, 4 and 8 weeks after infection. For the aerosol infection mice were infected with 30 viable CFU of H37Rv via the aerosol route using an inhalation exposure system (Glas-Col, Terre Haute, IN).

#### Lipopolysaccharide (LPS)/d-galactosamine (d-Gal)-induced septic shock

LPS (*Escherichia coli*, serotype O111:B4; Sigma, St Louis, MO) with d-Gal (Sigma) was injected intraperitoneally (i.p.) to induce systemic TNF and endotoxic shock as described previously.^32^ Briefly, mice were injected i.p. with 10 μg of LPS in combination with 20 mg d-Gal. Mice were monitored for 24 hr and the number of moribund mice was recorded.

#### Histology and immunohistochemistry

Lungs were removed and weighed. The organs were fixed in 4% buffered formalin, or frozen on dry ice and maintained at −80°. Two to 5 μm-thick sections of the lungs were cut and stained with either haematoxylin and eosin or Ziehl–Neelsen for acid-fast bacilli. Formalin-fixed, paraffin-embedded sections were de-paraffinized and re-hydrated through decreasing concentrations of alcohol as described.^7^ Sections were stained with a rabbit antimouse antibody specific for inducible nitric oxide synthase (iNOS; obtained from J. Pfeilschifter, University of Frankfurt, Germany). Sections were then washed in phosphate-buffered saline (PBS) and incubated for 30 min at room temperature with biotinylated rat anti-rabbit serum followed by Vectastain ABC system (Vector Laboratories Inc., Burlingame, CA) and 3,3′-diaminobenzidine (DAB) substrate. Finally, sections were mounted in Immunomount (Shandon, Pittsburgh, PA).

#### Colony-forming units (CFU)

Bacterial loads in the lung, liver and spleen of infected mice were evaluated at 2, 4 and 8 weeks post-infection. Organs were weighed and defined aliquots were homogenized in saline containing 0·04% Tween-80. Tenfold serial dilutions of organ homogenates were plated in duplicates onto Middlebrook 7H10 agar plates containing 10% OADC (Difco). Plates were incubated at 37° for 19–21 days and colonies counted. Data are expressed as mean CFU per organ.

#### Bronchoalveolar lavage

Under anaesthesia a 20-gauge catheter (Introcan, B. Braun, Melsungen AG, Germany) was inserted into the exposed trachea. The lungs were lavaged with two volumes of 300 μl PBS. Lavage fluid was centrifuged at 405 ***g*** for 5 min, the supernatant was removed, aliquoted and stored at −80° for cytokine and chemokine analysis. Lungs were subsequently lavaged with four volumes of 800 μl PBS. To remove traces of red blood cells, pooled samples were incubated in 1 ml red cell lysis buffer for 5 min, washed twice with 5 ml PBS. Samples were centrifuged at 405 ***g*** for 5 min and the cells were used for flow cytometric analysis. Procedures were performed under sterile conditions and samples kept on ice for the duration of the experiment.

#### Cytological analysis

Teflon coated, eight-well (6 mm) multi-well slides (Highveld Biological, Lyndhurst, South Africa) were used for cytospins. A 100 μl of a 2·5 × 10^5^cells/ml cell suspension was pipetted into each well and the liquid was drained from the well by attached Whatman filter paper. The slide was left to air dry overnight at room temperature. The cells were stained with RapiDiff staining kit, after removal of the filter paper, for differential cell counting. Unstained slides were stored at −20°, wrapped in aluminium foil. Cells were stained by immersing the slides three times (±5 s) in the following solutions: RapiDiff fixative, RapiDiff solution 1 (eosin in phosphate buffer) RapiDiff solution 2 (haematoxylin) and a final rinse in distilled H_2_O. Slides were left to dry for 1–2 hr at room temperature and mounted with entellan (Merck). Routinely, 100 cells per cytospin, in duplicate, per mouse were differentiated in a random fashion.

#### Flow cytometry

Cells (1 × 10^6^) were incubated with 25 μl fluorescence-activated cell sorting (FACS) blocking solution for 30 min after which they were washed with 1 ml FACS buffer and centrifuged at 405 ***g*** for 5 min. Cells were incubated with 2 μg/ml anti-CD3 phycoerythrin (PE; clone 145-2C11) and either 2 μg/ml anti-CD4 fluoroscein isothiocyanate (FITC; clone H129-19) or 2 μg/ml anti-CD8 FITC (clone 53-6-7) in a total volume of 50 μl for 30 min. Cells were washed with 1 ml FACS buffer, centrifuged at 405 ***g*** for 5 min and resuspended in FACS fixation buffer. Samples were kept on ice for the duration of the labelling procedure. Fluorescently labelled antibodies were purchased from BD Pharmingen (San Diego, CA). Samples were analysed on a FACSCalibur (Beckton Dickinson, San Jose, CA) flowcytometer using Cell Quest software.

#### Primary bone-marrow-derived macrophage (BMDM) cultures and infection

Mice 6–8 weeks old were killed and the femurs and tibia removed aseptically as described before by Müller *et al.*^33^ The bone marrows were flushed out with ice-cold DMEM (Dulbecco’s modified Eagle’s minimal essential medium with 4500 mg/l glucose, l-glutamine and 25 mm HEPES without sodium bicarbonate) using a syringe fitted with a 29-gauge needle. The cells eluted were resuspended in DMEM supplemented with 30% L929 conditioned medium and 20% horse serum and plated at a concentration of 2 × 10^6^cells/10 ml in Sterilin 90-mm bacteriological plates. On day 10, cells were pooled, pelleted by centrifugation and plated at 3 × 10^5^ cells/well in a 48-well plate (Costar; Corning Incorporated, Corning, NY). Cells were stimulated with 10 μg/ml LPS (*Escherichia coli*, serotype O111:B4; Sigma-Aldrich, St Louis, MO) and 100 U/ml rIFN-γ (BD Pharmingen) for 72 hr. In the infection study, the cells were prestimulated with 100 U/ml rIFN-γ for 16 hr and infected with *M. bovis* BCG (multiplicity of infection = 1:2) and the supernatants collected after 54 hr.

#### Enzyme-linked immunosorbent assay (ELISA) assay

TNF, IFN-γ and IL-12, IL-4, IL-5, monocyte chemoattractant protein-1 (MCP-1) and RANTES (regulated on activation, normal, T-cell expressed, and secreted) in supernatant of bronchoalveolar lavage fluid (BALF) were measured by ELISA with a sensitivity of <15 pg/ml (R&D Systems, Abingdon, UK and BD Pharmingen, San Diego, CA).

#### Statistical analysis

Statistical evaluation of differences between the experimental groups was determined by the use of the Student’s *t*-test and anova with a level of significance of *P* < 0·05.

## Results

### Absence of secreted TNF in TNF^tm/tm^ mice

**Figure 1:**
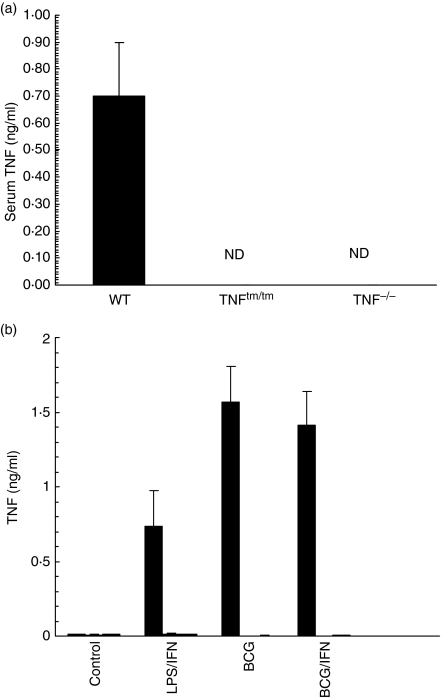
Absence of soluble TNF after endotoxin or mycobacterial stimulation. (a) *In vivo* injection of 100 μg LPS i.p. in TNF^tm/tm^ mice, TNF^−/−^ mice and WT mice, serum was taken at 90 min and analysed for TNF by ELISA. (b) LPS and BCG induced TNF at 4 hr in the supernatant of cultured macrophages. Data are expressed as the mean ± SD (*n* = 4 mice). ND, not detected.

To confirm that TNF^tm/tm^ knock-in mice do not secrete TNF, mice were injected i.p. with 100 μg LPS and blood was collected after 90 min and serum analysed by ELISA. In contrast to WT mice that displayed significant serum TNF levels, TNF was undetectable in the sera of both TNF^−/−^ and TNF^tm/tm^ mice (Fig. 1a). In addition, we showed that d-Gal sensitized WT mice were susceptible to LPS and succumbed from TNF-induced endotoxic shock after 24 hr as shown previously,^32^ whereas TNF^tm/tm^ mice survived the endotoxic shock as did the TNF^−/−^ mice (Table 1).

**Table 1:** Susceptibility of WT, TNF^tm/tm^ and TNF^−/−^ mice to LPS/d-Gal

Strain	Moribund mice
WT	5/5
TNF^tm/tm^	0/5
TNF^−/−^	0/5

WT, wild type; TNF, tumour necrosis factor.

We then investigated whether cultured BMDM could secrete TNF in response to LPS or after *M. bovis* BCG infection. Our results show that TNF was secreted by BMDM from WT mice, but undetectable in culture supernatants of BMDM from TNF^tm/tm^ and TNF^−/−^ mice (Fig. 1b).

Furthermore, membrane expression of TNF was found to be inducible upon stimulation.^25^ Taken together, these observations confirm the absence of secreted TNF in TNF^tm/tm^ mice as previously described.^25^

### TNF^tm/tm^ mice are more resistant to *M. bovis* BCG infection than TNF-deficient mice

**Figure 2:**
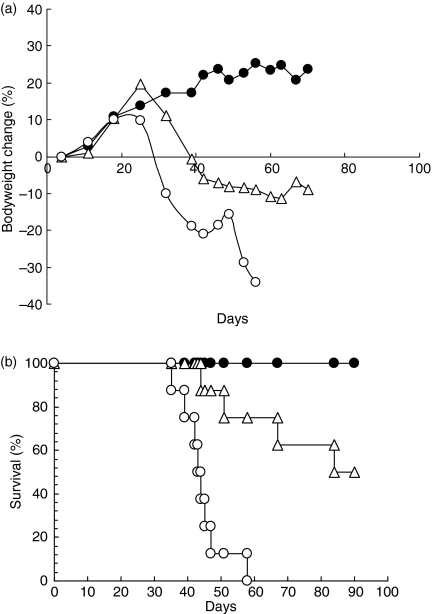
Survival of TNF^tm/tm^ and TNF^−/−^ mice infected with *Mycobacterium bovis* BCG. Body weight change (a) and survival (b) of TNF^tm/tm^ (Δ), TNF-deficient mice (○) and WT mice (•) infected i.v. with 2 × 10^6^ BCG bacilli and monitored for 3 months. Each group comprised 10 mice.

To determine whether membrane-bound TNF contributes to protective immunity, a comparative study was performed to determine the susceptibility of TNF^tm/tm^, TNF^−/−^ and WT mice to mycobacterial infection. Groups of 10 mice per strain were i.v. infected with 2 × 10^6^ CFU of the non-virulent vaccine strain *M. bovis* BCG and the rate of mortality was recorded. Within 3–4 weeks of infection TNF^−/−^ mice displayed rapid weight loss (Fig. 2a), impeded locomotor activity and succumbed to infection between 5 and 8 weeks (Fig. 2b). Although TNF^tm/tm^ mice manifested weight loss during the early phase of infection, the weights stabilized, and about 50% of the TNF^tm/tm^ mice survived the experiment. All WT mice survived the infection. From these observations, we concluded that membrane-bound TNF confers partial protection to systemic BCG infection in the absence of secreted TNF.

### Membrane TNF mice induce partial protective immunity to pulmonary *M. bovis* BCG infection

**Figure 3:**
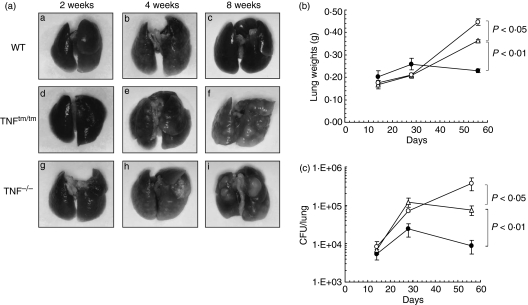
Lung pathology and uncontrolled bacterial growth in TNF^−/−^ mice. TNF^tm/tm^ mice (Δ), TNF^−/−^ mice (○) and WT mice (•) were infected i.v. with 2 × 10^6^ BCG *M. bovis* BCG. (a) Macroscopic lung changes in BCG infected mice at 2, 4 and 8 weeks post-infection. (b) Lung weights of BCG infected mice at 2, 4 and 8 weeks post-infection. (c) Bacterial load (CFU) was determined in lungs after 2, 4 and 8 weeks after BCG infection (2 × 10^6^ BCG i.v.). The results are expressed as the mean ± SD of five mice.

We and others have shown previously that the elimination of mycobacteria from the lung is absolutely dependent on TNF, while evidence for bacterial clearance from the liver and spleen in a TNF-dependent and -independent manner after systemic infection was reported.^3^,^4^,^7^ The importance of membrane-bound TNF for the control of mycobacterial growth in the lung was therefore compared in TNF^tm/tm^, TNF^−/−^ mice and WT mice infected i.v. with 2 × 10^6^ CFU *M. bovis* BCG. Macroscopic pathology of the lungs in TNF^−/−^ mice was characterized by the presence of large nodular lesions on the pleura at 4 weeks post-infection which augmented in size and became confluent at 8 weeks post-infection. Lesions were found also in TNF^tm/tm^ mice at 4 and 8 weeks post-infection, but were much smaller and more discrete, while WT mice displayed minor lesions (Fig. 3a). As a surrogate marker of inflammation we used lung weights. The lung weights were increased in both TNF^tm/tm^ and TNF^−/−^ mice at 8 weeks, while the lung weights of WT mice were unchanged (Fig. 3b). However, TNF^tm/tm^ mice had significantly lower lung weights than TNF^−/−^ mice (*P* < 0·05) suggesting partial protection from pulmonary infection.

We then determined the mycobacterial growth and elimination in the lungs over 8 weeks. All three mouse strains had similar bacilli levels (CFU) in the lungs 2 weeks after infection (Fig. 3c). At 4 weeks, WT mice displayed a small increase of CFU, but were thereafter able to eliminate the mycobacteria.

By contrast, bacilli burden was higher at 4 weeks in both TNF^tm/tm^ mice and TNF^−/−^ mice (*P* < 0·01) than in WT mice. TNF^−/−^ mice displayed uncontrolled mycobacterial growth with a significant increment in CFU in the lung at 8 weeks as compared to the 4 week values (*P* < 0·05). In contrast to TNF^−/−^ mice, TNF^tm/tm^ mice were able to control infection at 8 weeks. CFU in lungs of TNF^tm/tm^ at 8 weeks were lower than at 4 weeks (*P* < 0·05). In addition, CFU in the lungs of TNF^tm/tm^ mice were significantly lower than that of TNF^−/−^ mice (*P* < 0·05). Therefore, the data demonstrate that the sole presence of membrane-bound TNF is able to control *M. bovis* BCG infection in 50% of mice.

### Membrane TNF allows the expression of pulmonary granuloma

The establishment of granulomas is the manifestation of a vigorous cell-mediated immune response, which is crucial for inhibiting mycobacterial growth. A critical role of TNF for initiating and maintaining the structural integrity of granulomas has been demonstrated.^3^ We therefore asked whether membrane TNF is sufficient for granuloma formation upon *M. bovis* BCG infection.

**Figure 4:**
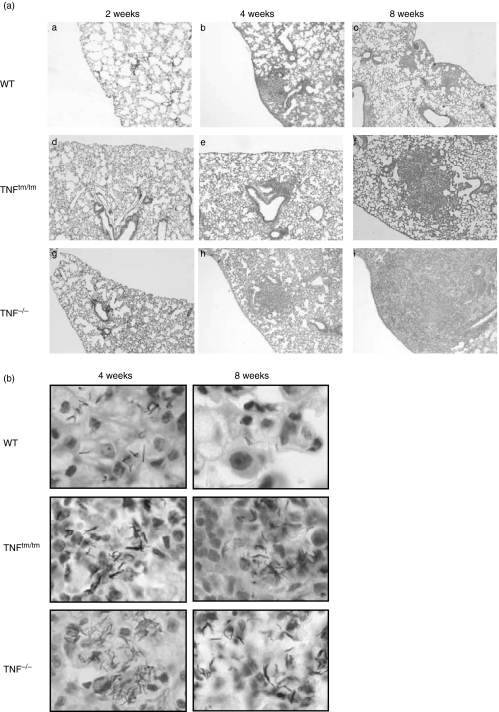
Histopathology in lungs of *M. bovis* BCG infected TNF^tm/tm^ mice, TNF^−/−^ mice and WT mice. Mice (five mice per group)were killed at 2, 4 and 8 weeks post-infection and the sections stained with haematoxylin and eosin (a) (400×) or with Ziehl–Neelsen (b) (1000×) to detect the acid-fast bacilli.

Pulmonary pathology in all three mouse strains at 2 weeks post-infection was characterized by areas that had normal alveolar and focal thickened septa, but the alveolar spaces were free from infiltrations (Fig. 4a) and there was no difference between the groups. At 4 weeks post-infection the lungs of WT mice displayed well-defined granulomatous lesions that were characterized by foamy epithelioid-like macrophages with surrounding and interspersed lymphocytes and prominent perivascular lymphocytic infiltration. At 8 weeks WT mice, which control *M. bovis* BCG infection, showed evidence of resolving granulomatous lesions.

As expected, TNF^−/−^ mice showed further thickening of septae containing inflammatory cells at 4 weeks, but did not develop granulomatous lesions and only sparse perivascular lymphocytic infiltration. At 8 weeks, a prominent perivascular and peribronchial lymphocyte infiltration was found in TNF^−/−^ mice. There was, however, no structural organization of the infiltrating lymphocytes and macrophages into a well-structured granuloma, and an increased recruitment of neutrophils was apparent.

In contrast, TNF^tm/tm^ mice displayed typical, but small granulomas at 4 weeks post-infection, which were less well demarcated than those found in WT mice. Epitheloid macrophages and lymphocytes were distinguishable and perivascular lymphocyte recruitment was evident. These early granulomas developed into enlarged pulmonary lesions at 8 weeks post-infection.

In addition, Ziehl–Neelsen staining revealed that bacilli in both WT and TNF^tm/tm^ mice are less abundant and predominantly confined to granulomas at 4 weeks and 8 weeks post-infection, whereas in TNF^−/−^ mice with the absence of properly defined granulomas the bacilli were dispersed in the tissue and predominantly extracellular (Fig. 4b). Therefore, in contrast to complete TNF deficiency the sole presence of membrane-bound TNF allowed a substantial granulomatous response with bactericidal properties.

### Membrane TNF allows partial cellular recruitment upon mycobacterial infection

**Figure 5:**
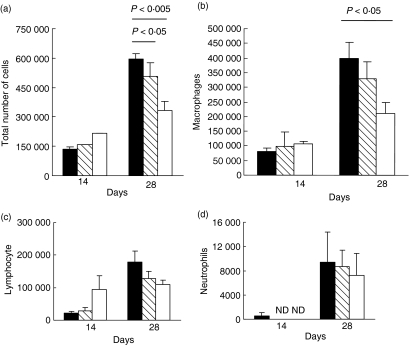
Cellular recruitment in bronchoalveolar space in the lungs of *M. bovis* BCG-infected TNF^tm/tm^ mice, TNF^−/−^ mice and WT mice. BALF was collected from TNF^tm/tm^ (line bar), TNF-deficient mice (open bar) and WT mice (black bar) infected i.v. with 2 × 10^6^ BCG bacilli at 2, 4 and 8 weeks. Cells (200 cells/mouse) were stained using a Rapidiff staining kit (Clinical Sciences Diagnostics, Southdale, South Africa), the average differential cell count were analysed in triplicate and recorded as previously described.^45^ Mean values ± SD of total cell counts (a), macrophages (b), lymphocytes (c) and neutrophils (d) are given (*n* = 5).

In view of the granulomatous response elicited in TNF^tm/tm^ mice after *M. bovis* BCG infection we investigated the cellular recruitment in the lungs. Cells from BAL were obtained at 2 and 4 weeks post-infection. WT mice showed significant recruitment of macrophages, lymphocytes and neutrophils into the BALF 2 and 4 weeks after infection. The total cell number was highest in WT mice, followed by TNF^tm/tm^ and TNF KO mice, which had significantly reduced total cell recruitment (*P* < 0·05) at 4 weeks (Fig. 5a). The macrophage recruitment in BALF was significantly reduced in TNF^tm/tm^ and TNF KO mice (*P* < 0·05) (Fig. 5b), while the lymphocyte counts (Fig. 5c) showed only a trend of reduction and the neutrophils (Fig. 5d) were elevated, but not different among the experimental groups at 4 weeks.

**Figure 6:**
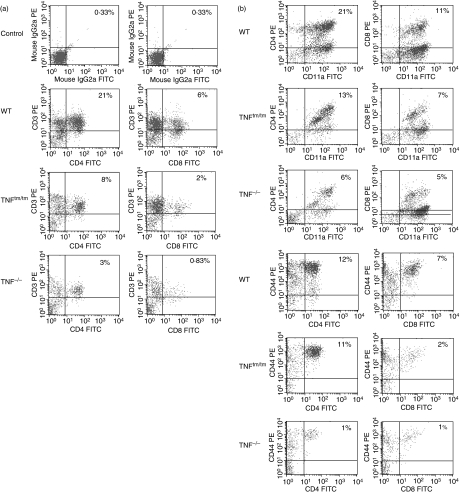
Reduced pulmonary recruitment of CD4 and CD8 T cells in TNF^tm/tm^ mice and TNF^−/−^ mice. BALF was collected from *M. bovis* BCG-infected TNF^tm/tm^, TNF^−/−^ and WT mice at 4 weeks post-infection as described in *Methods*. Four mice were individually analysed for each mouse strain. (a) Recruitment of CD4 and CD8 T cells in WT, TNF^tm/tm^ and TNF^−/−^ mice. (b) Expression of CD11a and CD44 on CD4 and CD8 cells in WT, TNF^tm/tm^ and TNF^−/−^ mice.

CD4^+^ and CD8^+^ T cells are critical for the generation of cell-mediated immunity. We therefore investigated whether membrane-bound TNF is sufficient to recruit specific T-cell subsets. Cells from BAL at 4 weeks post-infection were analysed by flow cytometry. In the presence of both soluble and secreted TNF (WT mice), effective recruitment of CD3^+^ CD4^+^ T cells and CD3^+^ CD8^+^ T cells occurred (Fig. 6a). In the absence of TNF, T-cell recruitment was reduced sevenfold (CD3^+^ CD4^+^ T cells: 21% versus 3% and CD3^+^ CD8^+^ T cells: 6% versus 0·8%). By contrast, the sole expression of membrane-bound TNF was sufficient to allow a significant recruitment of CD4 and CD8 T cells in BALF of TNF^tm/tm^ mice, which was only 2–3 × lower than in WT mice (CD3^+^ CD4^+^ T cells: 21% versus 8% and CD3^+^ CD8^+^ T cells: 6% versus 2%).

In addition, we investigated the activation status of the recruited T cells. We found that >95% of the cells recruited were activated, as defined by the high expression of CD11a and CD44, and there was no difference among the three groups (Fig. 6b).

**Figure 7:**
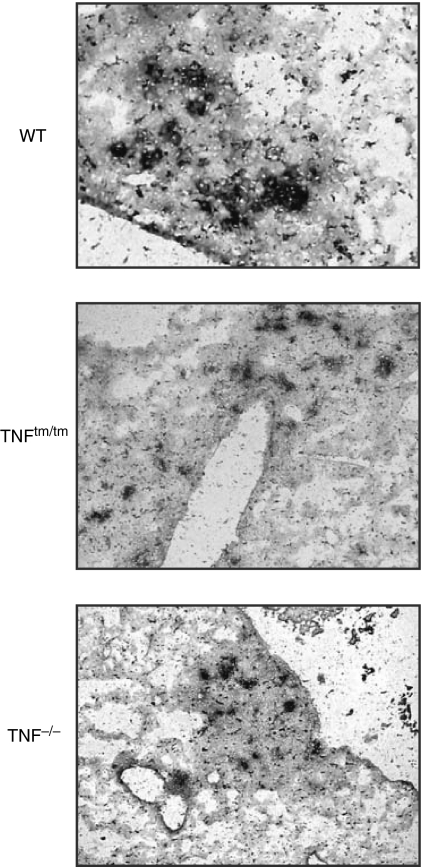
Reduced expression of iNOS in lungs of BCG-infected TNF^tm/tm^ mice and TNF^−/−^ mice at 4 weeks post-infection. Lung sections were incubated with iNOS antibody as described. Magnification 100×.

As the T-cell recruitment was reduced in BAL, we asked whether the inflammatory response in the lungs of BCG-infected mice differed in the total or partial absence of TNF. As shown in Fig. 4, TNF^tm/tm^, but not TNF^−/−^ mice were able to recruit abundant lymphocytes and macrophages into the lung tissue allowing the formation of granulomas, which were, however, less well developed than in WT mice. We asked whether the partial recruitment of lymphocytes as shown in BALF was sufficient to support macrophage activation. We measured iNOS as an indicator of macrophage activation, semiquantitavely and observed expression in TNF^tm/tm^ mice, but at a lower level compared with WT mice. However, iNOS expression in TNF^tm/tm^ mice was substantially higher than in TNF ^−/−^ mice (Fig. 7).

These data suggest that membrane TNF allows substantial recruitment of T cells and the activation of macrophages resulting in mycobactericidal effector mechanisms, which are largely absent with complete TNF deficiency.

### Membrane TNF enables cytokine and chemokine secretion

We next investigated the secretion of cytokines and chemokines in BALF as possible factors that influences cellular recruitment and protective immunity. Chemokine expression and hence cell movement have been shown to be in part TNF dependent.^14^,^34^–^36^ We therefore investigated the contribution of membrane-bound TNF in the generation of the T helper 1 (Th1)-defining cytokines, IFN-γ and IL-12, the Th2 cytokines, IL-4 and IL-5, and the monocyte and lymphocyte chemoattractants MCP-1 and RANTES in BAL.

**Figure 8:**
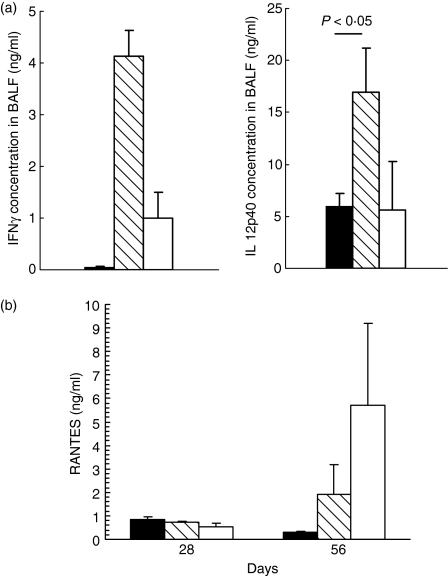
Cytokine and chemokine secretion in BALF of TNF^tm/tm^ mice (line bar), TNF^−/−^ mice (open bar) and WT mice (black bar). (a) IFN-γ and IL-12 were measured at 28 days, and (b) RANTES was analysed at 28 and 56 days. The results are expressed as the mean ± SD (*n* = 4).

Levels of IL-12 at 4 weeks post-infection were significantly higher in TNF^tm/tm^ mice in comparison with WT mice or TNF^−/−^ mice (Fig. 8a). IFN-γ secretion was low in WT mice and highest in TNF^tm/tm^ mice (Fig. 8a). As expected, no TNF could be detected in TNF^tm/tm^ and TNF^−/−^ mice and MCP-1 was undetectable at 4 weeks (not shown). RANTES levels peaked at 4 weeks post-infection in WT mice and were significantly (*P* ≤ 0·01) reduced at 8 weeks post-infection in WT mice (Fig. 8b). TNF^tm/tm^ and TNF^−/−^ mice displayed elevated levels at 8 weeks post-infection (*P*≤ 0·01). We were unable to detect the secretion of IL-4 or IL-5 during any stage of the infection.

The high level of IFN-γ and IL-12 at 4 weeks, and RANTES at 8 weeks post-infection in TNF^tm/tm^ mice suggest a strong Th1 response, which was confirmed by an enhanced cutaneous delayed-type reaction in infection in both TNF^tm/tm^ and TNF^−/−^ mice (data not shown). The higher levels of RANTES in TNF^−/−^ mice might be related to the high bacterial burden in the lung.

### Membrane TNF generates partial protective immunity against *M. tuberculosis* aerosol infection

**Figure 9:**
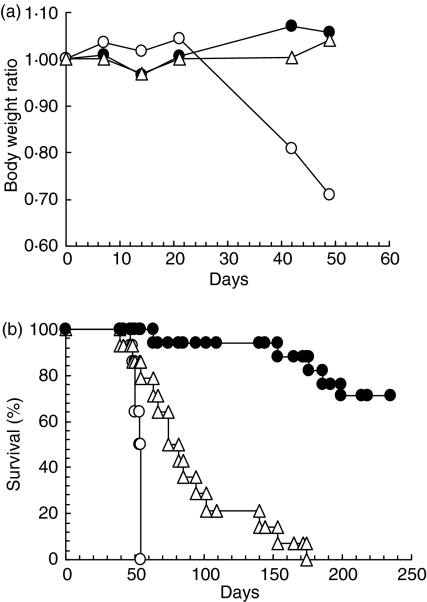
Body weights and survival of TNF^tm/tm^, TNF^−/−^ and WT mice exposed to *Mycobacterium tuberculosis* infection*.* TNF^tm/tm^ mice (Δ), TNF^−/−^ mice (○) and WT mice (•) were infected by aerosol (10–30 CFU/lung), and the survival monitored over 8 weeks. Bodyweight change is expressed as a ratio, relative to the bodyweight measured on the day of infection. Each group comprised 10 mice.

In order to determine whether the partial protective immune response that was generated after the infection with *M. bovis* BCG is applicable during a virulent infection, mice were infected with a low dose (10–30 CFU/lung) of *M. tuberculosis* H37Rv by aerosol inhalation. Body weight ratios were determined for 7 weeks of infection and the groups were scored for mortality. Progressive infection was paralleled by continual lowering of body weight ratios and mortality in TNF^−/−^ mice (Fig. 9a,b). In contrast, WT mice and TNF^tm/tm^ did not show a loss of body weight during the early course of infection. After 8 weeks of infection, ∼80% of TNF^tm/tm^ mice were still alive, while all TNF^−/−^ mice had succumbed to fatal pulmonary tuberculosis.

**Figure 10:**
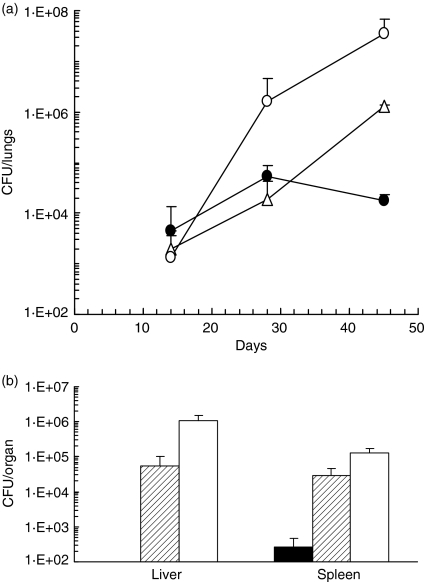
Mycobacterial burden in TNF^tm/tm^, TNF^−/−^ and WT mice exposed to *M. tuberculosis* infection. (a) Lung (14, 28 and 45 days), (b) liver and spleen (45 days). TNF^tm/tm^ (Δ, line bar), TNF-deficient mice (○, open bar) and WT mice (•, black bar) were infected by aerosol (10–30 CFU/lung). The results are expressed as the mean ± SD (*n* = 5).

We further assessed the mycobacterial burden in the lung of *M. tuberculosis*-infected mice. While TNF^−/−^ mice displayed an uncontrolled infection with significantly increased CFU already at 4 weeks (*P* < 0·05), the TNF^tm/tm^ mice showed at least a partial control of mycobacterial growth, with CFU values significantly less (*P* < 0·05) than TNF^−/−^ mice at 45 days (Fig. 10a). CFU counts in liver and spleen, as a measure of the systemic dissemination of *M. tuberculosis*, were significantly higher in TNF^tm/tm^ and TNF^−/−^ mice 45 days post-infection than in WT (Fig. 10a). However, the hepatic CFU in TNF^tm/tm^ mice were lower (*P* < 0·05) than that of TNF^−/−^ mice.

**Figure 11:**
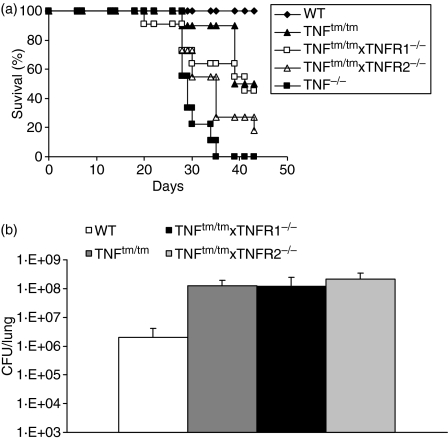
Comparison of resistance to *M. tuberculosis* infection in TNF^tm/tm^ and TNFR1^−/−^ or TNFR2^−/−^ × TNF^tm/tm^ double transgenic mice. (a) Survival of mice infected by H37Rv i.n. (10–30 CFU per lung, *n* = 9–11 mice pooled from two independent experiments). (b) Mycobacterial burden in the lung (CFU) 6 weeks after infection. The results are expressed as the mean ± SD (*n* = 4–6 mice per group of transgenic mice).

Lastly, we asked whether the protective effect of membrane TNF is signalled through TNFR1 or TNFR2. Therefore, we generated double transgenic mice by crossing TNF^tm/tm^ mice with TNFR1^−/−^ or TNFR2^−/−^ mice. We further assessed the mycobacterial burden in the lung of *M. tuberculosis* infected mice. In this experiment, TNF^−/−^ mice lost weight rapidly and died within 5 weeks, while TNF^tm/tm^ mice were less sensitive, half surviving up to 45 days. TNF^tm/tm^ × TNFR2 KO mice seemed essentially as sensitive as TNF^−/−^ mice, while TNF^tm/tm^ × TNFR1 KO mice behaved more like TNF^tm/tm^ mice (Fig. 11a). Therefore, signalling of membrane TNF through TNF-R2 likely confers protection. This hypothesis is further corroborated by increased mycobacterial loads in the lung of TNF^tm/tm^ × TNFR2 KO at 6 weeks post-infection, a time point when all TNF^−/−^ had succumbed, essentially doubled as compared to TNF^tm/tm^ or TNF^tm/tm^ × TNFR1 KO mice (Fig. 11b).

Therefore, the sole expression of membrane TNF provided partial protection against H37Rv infection and the data using the double transgenic mice indicate that membrane TNF signalling through TNF-R2 confers the protective effect of membrane-bound TNF.

## Discussion

Here we demonstrated that the Δ1-12 TNF mutant,^25^ which expresses only membrane TNF is partially resistant to infection to the non-virulent vaccine strain *M. bovis* BCG with 50% of mice surviving the experimental period, while all TNF-deficient mice succumb to infection. TNF^tm/tm^ mice were able to recruit and activate macrophages and T cells and generate mycobactericidal granuloma in response to BCG infection. However, resistance to the virulent *M. tuberculosis* strain was less pronounced, suggesting substantial differences between this and the Δ1-9,K11E mouse mutant.^24^ This TNF mutant differs from the Δ1-9,K11E TNF allele^24^ in several aspects – the lymphoid structure especially is abnormal. We and others demonstrated that the Δ1-9,K11E mutant has enhanced resistance to *M. tuberculosis* infection^26^,^27^ and *Listeria* infection.^28^

A critical role of TNF for the effective control and resolution of mycobacterial infection has been demonstrated previously.^4^,^7^,^35^ Further, TNFR1-mediated signalling is required for generating protective immunity against mycobacterial infection,^5^ which is probably more important than TNFR2 signalling.^11^ TNF provided by recombinant BCG expressing TNF may reconstitute granuloma formation and host response in TNF^−/−^, but not in TNFR1^−/−^ mice, demonstrating the critical role of TNF and TNFR1 signalling.^37^

The partial protection that is generated in TNF^tm/tm^ mice could indicate local cell-to-cell TNF signalling by membrane expressed TNF on T cells or macrophages at the site of infection leading to a partial activation of the immune cells. Several biological functions of membrane TNF have been reported previously and a preferential TNFR2 signalling has been suggested *in vitro*^19^,^38^ and *in vivo*^21^,^38^,^39^ using transgenic mice expressing membrane TNF. The present data therefore suggest a functional role of TNFR2 in host protection. Furthermore, membrane TNF has been shown to be involved in reverse (outside-to-inside) signalling. Upon ligation of its receptor membrane-bound TNF-expressing cells are activated to express E-selectin.^40^ Thus, membrane-bound TNF, at least in T cells, might function as a bipolar positive regulator of inflammation either transmitting signals as a ligand to target cells or receiving signals through membrane TNF itself into T cells.

Although the exact mechanism of how protection is acquired through membrane TNF is unclear, membrane TNF on activated T cells might be sufficient for partial activation of macrophages; this results in the upregulation of iNOS expression, which is crucial for bacterial killing.^21^,^41^–^43^ Membrane TNF expressed on T cells might replace secreted TNF to form granulomas in TNF^tm/tm^ mice. Macrophage and lymphocyte recruitment was significantly reduced in TNF^−/−^ mice, with a significant reduction of the CD3^+^ CD4^+^ and CD3^+^ CD8^+^ T-cell subsets in the absence of TNF, which might be related to reduced chemokine production in the absence of TNF.^14^ As the recruitment of the different cell types was partially restored in TNF^tm/tm^ mice, membrane TNF on T cells or other cells might provide a partial signal for chemokine induction and hence increased cell trafficking. The contribution of CD4^+^ T-cells in cell-mediated immunity against mycobacteria has been investigated by the use of neutralizing antibodies and gene-deficient mice.^1^ In the absence of CD4^+^ typical granuloma formation in T cells was largely absent.^44^

However, in view of the only partial activation and protection, which is likely caused by direct cell–cell contact, secreted TNF and hence distal signalling, appears to be required for a full protective host response. This finding concurs with the recent data demonstrating reduced severity of autoimmune encephalomyelitis, but little effect on inflammation in the spinal cord in a similar genetic mouse model.^24^

Lastly, the infection studies in double TNF^tm/tm^× TNFR1 or R2^−/−^ transgenic mice rescued protection in TNF^tm/tm^ × TNFR2^−/−^ mice and comparable protection in TNF^tm/tm^ × TNFR1^−/−^ mice as in TNF^tm/tm^ mice suggest that membrane signalling though TNFR2 likely provides host protection. Our data in this infection model using the Δ1-12 TNF mutant mouse shows a reduced protective immune response during mycobacterial infection when compared to previous findings in which a membrane TNF transgenic mutant or the Δ1-9,K11E mutant were used. Our findings indicate that membrane TNF, in the Δ1-12 TNF mutant may preferentially induce protection through TNFR2. Receptor signalling in membrane TNF transgenic mutant or the Δ1-9,K11E mutant may be mediated through both TNFR1 and TNFR2 different, accounting for the differences observed in the degree of protection afforded by membrane TNF in the different mutant strains.

The data suggest that sparing membrane expressed TNF in therapeutic interventions neutralizing TNF such as in rheumatoid arthritis might reduce infectious complications. In summary, we show that membrane TNF participates in cell-mediated immunity against mycobacterial infection. The protective effect of membrane-bound TNF is likely mediated through TNFR2 signalling.
